# Electrical behavior of MIS devices based on Si nanoclusters embedded in SiO_*x*_N_*y *_and SiO_2 _films

**DOI:** 10.1186/1556-276X-6-170

**Published:** 2011-02-24

**Authors:** Emmanuel Jacques, Laurent Pichon, Olivier Debieu, Fabrice Gourbilleau

**Affiliations:** 1 Groupe Microélectronique, IETR, UMR CNRS 6164, Campus de Beaulieu, Rennes Cedex, 35042 France; 2 Centre de Recherche sur les Ions, les MAté riaux et la Photonique, UMR CEA/CNRS/ENSICAEN/UCBN 6 boulevard du Maréchal Juin, Caen Cedex 4, 14050 France

## Abstract

We examined and compared the electrical properties of silica (SiO_2_) and silicon oxynitride (SiO_*x*_N_*y*_) layers embedding silicon nanoclusters (Sinc) integrated in metal-insulator-semiconductor (MIS) devices. The technique used for the deposition of such layers is the reactive magnetron sputtering of a pure SiO_2 _target under a mixture of hydrogen/argon plasma in which nitrogen is incorporated in the case of SiO_*x*_N_*y *_layer. Al/SiO_*x*_N_*y*_-Sinc/p-Si and Al/SiO_2_-Sinc/p-Si devices were fabricated and electrically characterized. Results showed a high rectification ratio (>10^4^) for the SiO_*x*_N_*y*_-based device and a resistive behavior when nitrogen was not incorporating (SiO_2_-based device). For rectifier devices, the ideality factor depends on the SiO_*x*_N_*y *_layer thickness. The conduction mechanisms of both MIS diode structures were studied by analyzing thermal and bias dependences of the carriers transport in relation with the nitrogen content.

## Introduction

Silicon heterojunctions have been extensively studied for the understanding of the physics of the device as well as their applications to majority of the carrier rectifier [1], photodetectors [2], solar cells [3], and indirect gap injection lasers [4]. Because of its indirect band gap, silicon is a highly inefficient material for a light emitter. However, to overcome this problem, different approaches were developed in this last decade for the fabrication of Si-based light emitting sources made of silicon nanoclusters (Sinc) embedded in silica or silicon oxynitride (SiO_2_-Sinc or SiO_*x*_N_*y*_-Sinc) matrix.

Due to quantum confinement effects, Sinc are characterized by an energy band gap which is enlarged with respect to bulk silicon and by an intense room temperature photoluminescence that can be obtained in the visible-near infrared (IR) range [5,6]. Previous study [7] recently reported that the presence of incorporated nitrogen species influences the silicon nanoclusters' growth and affects the photoluminescence of the SiO_*x*_N_*y*_-Sinc layer. In addition, IR light emitting properties were also reported in matrix embedding Sinc and rare earth [8-11]. In such system, the emitting rare earth ions benefit from the quantum confinement properties of the carriers generated within Sinc to be efficiently excited by an energy transfer mechanism. Electroluminescence of SiO_*x*_N_*y*_-Sinc-based IR light emitting devices is limited by the difficulty in carrier injection. Therefore, prior to developing IR light emitting devices, a good understanding of optimum carrier injection in SiO_*x*_N_*y*_-Sinc type layers is needed. In this way, previous works have been recently reported on electrical properties in metal-insulator-semiconductor (MIS) devices fabricated with such silicon-rich oxide layers either deposited by (1) plasma-enhanced chemical vapor deposition technique [12] or by (2) magnetron co-sputtering of Si, SiO_2 _[13]. In the first case, rectifying behavior was observed, but not in the second. In addition, thermal dependence of the carrier transport was not studied.

In this present work, we report a detailed study of the carrier transport governing electrical properties of SiO_2_-Sinc and SiO_*x*_N_*y*_-Sinc layers integrated in MIS devices. Layers are deposited by reactive magnetron sputtering of a pure SiO_2 _cathode. The thermal and the bias dependences of the carrier transport are analyzed. The aim of such study consists in fabricating a thin layer for future electroluminescent devices doped with rare earth ions. Thus, one of the key parameters is to overcome the insulating characteristic of the SiO_2 _matrix by incorporating nitrogen.

### Device technology and experimental details

**Figure 1:**
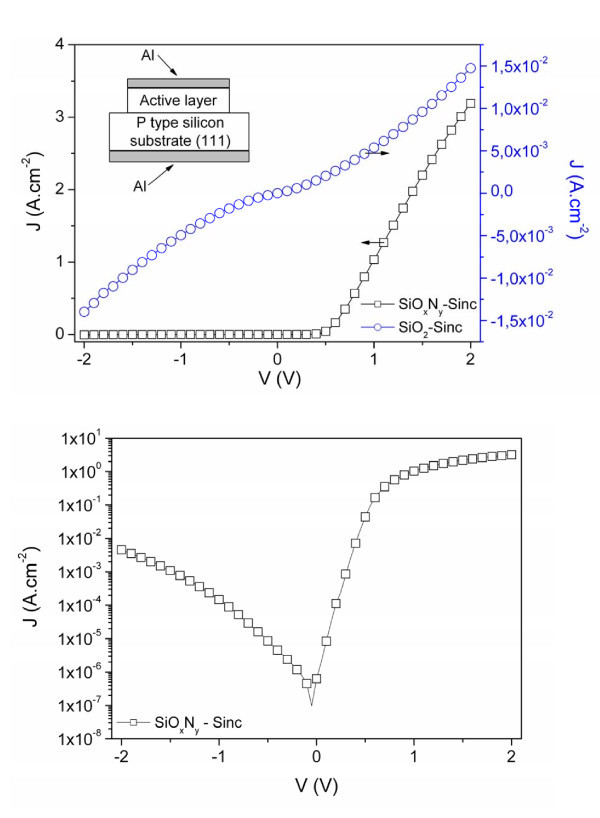
**Comparison of devices**. **(a) **Linear current density-voltage characteristics of MIS structure based on SiO_2_-Sinc and SiO_*x*_N_*y*_-Sinc layer. Inset: Schematic cross section of the tested MIS structures, **(b) **current density-voltage characteristics of MIS structure based on SiO_*x*_N_*y*_-Sinc layer plotted in semi log scale.

Devices are elaborated on p-type (111)-oriented silicon substrates with resistivity in the range 0.001-0.005 Ω cm (see inset of the Figure 1a). A SiO_2_-Sinc (or SiO_*x*_N_*y*_-Sinc) layer was deposited by reactive magnetron sputtering of a pure SiO_*2 *_target under a mixture of hydrogen/argon plasma. In the case of SiO_*x*_N_*y *_layer, nitrogen gas is incorporated in the plasma leading to a final concentration of 15 at.% in the film. For more details on the deposition process, see ref. [14]. Two thicknesses d of 30 and 65 nm were deposited for each layer, which was subsequently submitted to an optimized annealing treatment at 1,050°C during 30 min under N_2 _flux. Next, aluminum was thermally evaporated on active layer. Both aluminum and active layers were patterned by wet etching to define the geometry of the device. A second thermal evaporation of aluminum on the back surface was carried out to ensure the ohmic contact with the p-type crystalline silicon. Finally, the devices were annealed into forming gas (H_2 _to N_2_, 10%) at 390°C during an optimum duration of 30 min to stabilize the electrical properties of the devices.

Static electrical characteristics *J*(*V*) are collected by using an HP 4155B semiconductor parameter analyzer. For temperature measurements from -70°C to 230°C samples are placed in a cryostat under vacuum (10^-5^-10^-4 ^Pa). All measurements were made in darkness on more than 20 devices homogeneously located over the 2-in. surface substrate. The area of each tested device is 0.32 mm^2^.

## Results

The comparison of the devices' *J*(*V*) characteristics of SiO_*x*_N_*y*_-Sinc and SiO_2_-Sinc-based devices reveals a high rectifying behavior for the former while no rectifying behavior is observed for the latter (Figure 1a). *J*(*V*) electrical characteristics for SiO_2_-Sinc layer is typical of conduction in (semi) insulating material. Such a result suggests that the rectifying behavior of the SiO_*x*_N_*y*_-Sinc layer could be explained by the presence of the incorporated nitrogen acting as N-type doping specie [15]. In this case, at low forward bias (0 V ≤ V ≤ 0.8 V), current density taking account serial resistance can be described by:

(1) $$J=J_{0}\left(\exp\frac{qV-RJ}{nk_{B}T}-1\right)$$

where *q *is the electron charge, *k*_B _the Boltzmann's constant, *n *the ideality factor dealing with current dominated by carrier diffusions (*n *= 1) and/or by carriers recombination processes at defects (*n *= 2), *R *the global serial resistance and *J*_0 _the saturation current density. Ideality factor *n *and serial resistance *R *are deduced by fitting our experimental results with the theoretical model (1). The resistance *R *is likely to arise from minority carrier space charge, the bulk resistances, and finally contact resistance. In our case, *n *was estimated to *n *≈ 1.2 indicating that carrier injection is dominated by the carrier diffusion process. For such N-rich devices *J*(*V*) plots have an excellent rectifying ratio (> 10^4 ^at V = ± 1 V) (Figure 1b) leading to a higher injected current level than reported in the literature [16-18]. In addition, at high voltages (2 V > V > 0.8V), current deviates from the exponential behavior due to the low global resistance series (20 Ω <*R *< 40 Ω).

Studies of bias and temperature dependence of the electrical properties are carried out to analyze the carrier transports for the two devices. Several models are currently used to understand the carrier injection. For SiO_*x*_N_*y*_-Sinc-based devices biased in the reverse mode, the Poole-Frenkel (PF) model is the most convenient. In this approach, electrons are considered to be thermally emitted from the randomly distributed traps to the conduction band as a result of the lowering of the columbic potential barrier by an external electric field. This model is described by the following relation [19]:

(2) $$J=N\mu E\exp\left(-\frac{\varphi_{0}}{k_{B}T}\right)\exp\left(\frac{\beta_{PF}E^{1/2}}{k_{B}T}\right)$$

where *J *is the current density, *N *the density of trapping sites, *μ *the effective carrier mobility, *E*(*= V/d*) the local electric field, *Φ*_0 _the zero field trapped energy barrier depth and *β*_PF _the PF coefficient.

**Figure 2:**
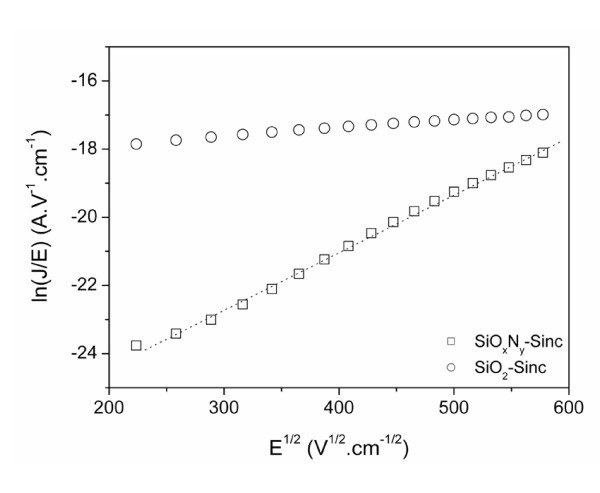
**Plots of the ln(*J*/*E*) vs *E***^**1/2 **^**of Al/SiO**_***x***_**N**_***y***_**-Sinc/p-Si and Al/SiO**_**2**_**-Sinc/p-Si structures**.

This latter is extracted from the linear representation of ln(*J*/*E*) = *f*(*E*^1/2^) (Figure 2) and is related to the permittivity of active layer, and thus to the material composition, following the relation:

(3) βPF=(q3πε0εr)12

The permittivity obtained is also compared to the value deduced from quasi static C-V measurements. The coefficients deduced from the PF relation provides *ε*_r _= 5.6 and *ε*_r _= 4.4 for the 30-nm and the 65-nm-thick SiO_*x*_N_*y*_-Sinc layers, respectively while from C-V measurements, the corresponding permittivity obtained are *ε*_r _= 5.1 and *ε*_r _= 4.3. Such similar results are consistent with values obtained with the PF model to explain the reverse current behavior (V < -0.2 V). The difference of permittivity noticed could be explained either by a change of the density of Si nanoclusters (for the same Si content) or by a modification of the Si excess with the thickness. Considering that we observe an increase of the refractive index from 1.61 to 1.75 for 65 nm and the 30-nm layer thicknesses respectively, it suggests that during the first step of the deposition process, the starting growth process conditions could promote the incorporation of Si within a few nanometres thick due to the vicinity of the Si substrate.

Temperature measurements of the reverse current have been carried out in order to confirm the Poole-Frenkel mechanism for which the current is thermally activated. Thus, the corresponding activation energy *E*_a _is defined by the following relation:

(4) Ea=φ0−βPFE12

**Figure 3:**
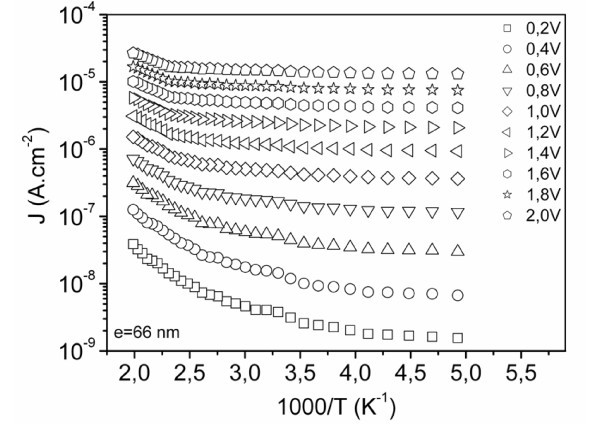
**Arrhenius representations of the current density of the SiO**_***x***_**N**_***y***_**-Sinc layer made MIS structures**.

Arrhenius diagrams of ln(*J*) vs 1/*T *(Figure 3) show a temperature dependence of the reverse current that confirms a thermal activation of the current and consequently that the carrier transport follows a PF mechanism. The carriers' emission from defects following such a mechanism is enhanced by a barrier lowering where the electrical field is the most important, that means at the PN junction interface. In this way, the Poole-Frenkel emission of carriers may likely occur from defects in the bulk of the SiO_*x*_N_*y *_matrix located at Sinc/SiO_*x*_N_*y *_interfaces [12] close to the p-type silicon substrate.

**Figure 4:**
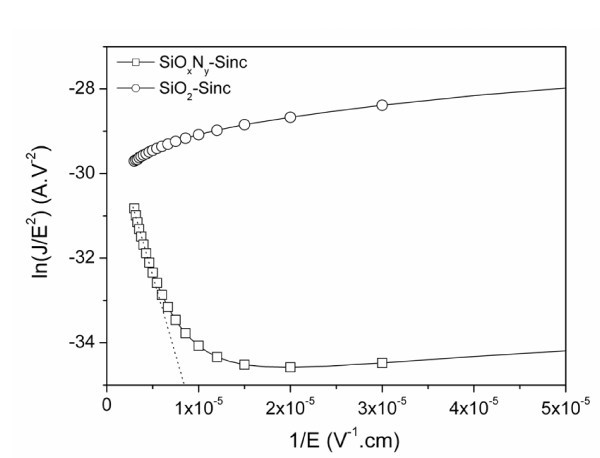
**Fowler-Nordheim tunnel representation of Al/SiO**_***x***_**N**_***y***_**-Sinc/p-Si and Al/SiO**_**2**_**-Sinc/p-Si structures**.

For low temperatures, *J *does not depend on the temperature indicating that it is more representative of a tunnel conduction way. This behavior is more pronounced at high reverse bias because of the linear decrease of the *J*/*E*^2 ^= *f*(1/*E*) plot (Figure 4) according to the Fowler-Nordheim model given as [20]:

(5) J=CE2exp(−42m*(ΦB)323qhE)

where *C *is a constant both depending on the elementary charge *q*, the barrier height *Φ*_B, _and the Planck's constant *h*, and where m* stands for the carriers' effective mass. This conduction mechanism could be effective between the silicon nanoclusters through the silicon oxynitride.

**Figure 5:**
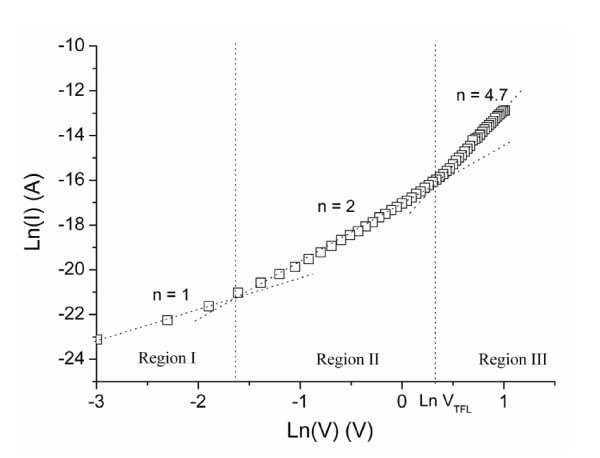
**Ln *I *vs. ln *V *representation of MIS structure based on SiO**_**2**_**-Sinc layer**.

The same approach has been used for the N-free Si-rich layer. No current variation in the SiO_2_-Sinc layer according to the electric field has been observed (Figure 2) demonstrating that Poole-Frenkel mechanism is not the conduction mode in such an MIS structure. Fowler-Nordheim tunneling conduction has also been tested for this layer (see Figure 4) and no variation is observed following equation 5. Therefore, the electrical behavior of this layer in both reverse and forward bias cannot be explained by Fowler-Nordheim tunneling conduction. However, the forward current characteristics plotted with a representation in ln(*I*) = ln(*V*) in Figure 5 can be divided into three regions. In this case, the current follows a voltage power law (*I *∝ *V*^n^). In the first region (V < 0.2 V) *n *= 1 corresponding to an ohmic behavior where the deduced intrinsic resistivity of the material (*ρ *= 2.7 × 10^11 ^Ω cm^-1^) is typical of (semi) insulator. For higher voltages (0.2 V < V < 0.4 V), conduction mechanism is due to space charge limited conduction dominated by a discrete trapping level in the second region (*n *= 2) and by exponential distribution in the third region (*n *> 2). From this characteristic, the density *n*_t _of the trapped electrons can be extracted accordingly:

(6) $$n_{t}=\frac{2\varepsilon \varepsilon_{0}}{qd^{2}}V_{TFL}$$

where *ε *is the permittivity of the material and *V*_TFL _is the voltage at which the current significantly increases. The permittivity extracted from C-V measurements is *ε*_r _= 3.95. *V*_TFL _is defined as the intersection between the linear parts of the second and the third region. The value of the trap density *n*_t_, acting as quality factor of the SiO_2_-Sinc layer, has been estimated to be 5.39 × 10^16 ^cm^-3^. The presence of trap centers could be associated to the density of Si nanoclusters in the silicon oxide matrix as it has been reported in a previous work [21]. In the case of SiO_*x*_N_*y *_layer, as previously discussed, conduction mechanism appears to be different. In such a layer, the nitrogen is suspected to passivate the trap centers close to the silicon nanoclusters and thus promoting N-type doping effect responsible of pn junction creation between the active layer and the P-doped silicon substrate.

## Conclusion

Conduction mechanisms of SiO_2_-Sinc and SiO_*x*_N_*y*_-Sinc layers have been studied and compared. The use of silicon oxynitride with embedded silicon nanoclusters has been validated in order to achieve diode with high rectifying behavior. Nitrogen significantly modifies the electrical behavior of the layer. It is suspected to be both responsible of a of (1) a defect passivation at the interface of silicon oxide matrix and silicon nanoclusters and (2) to act as N-doping specie and to promote a pn junction creation between active layer and P-doped silicon substrate.

This study has shown the interest to use nitrogen in silicon matrix with silicon nanoclusters to improve the current injection in the MIS structure. This effect could be interesting for an energy transfer to the rare earth ions for an infrared emission in such structures based on silicon-rich oxynitride layer doped with rare earth.

## Competing interests

The authors declare that they have no competing interests.

## Authors' contributions

EJ fabricated and electrically characterized the devices, he also analysed the conduction mechanisms of the devices and wrote a part of the manuscript. LP analysed and explained the conduction mechanisms in the device and participated in the writing of the manuscript. OD fabricated the thin films and carried out the optical and microstructural characterizations. FG conceived of the study and participated in the coordination of the project. All authors read and approved the final manuscript.
